# Supplementary material for: Altered microRNA Transcriptome in Cultured Human Liver Cells upon Infection with Ebola Virus
Source: Int J Mol Sci. 2021 Apr 6;22(7):3792. doi: 10.3390/ijms22073792 (PMC8038836; doi:10.3390/ijms22073792)
Supplement: Supplementary file 1 [file ijms-22-03792-s001.zip › Supplementary_File/C_ GO_Analysis_Results/16-30nt_go_Reston-24h-Huh7_vs_Control-24h-Huh7_down.mature_mirna_targets/MF_result(Human).html]

| GO.ID | Term | Ontology | Count | Pop.Hits | List.Total | Pop.Total | Fold.Enrichment | Pvalue | FDR | Enrichment.Score | Gene.Ratio | GENES |
| --- | --- | --- | --- | --- | --- | --- | --- | --- | --- | --- | --- | --- |
| GO:0060090 | molecular adaptor activity | Molecular function | 9 | 191 | 178 | 17548 | 4.64533207835755 | 0.000147293494873077 | 0.190892369355508 | 3.83181643308345 | 0.050561797752809 | VAV3//ARHGAP1//CNTNAP1//MAPK8IP1//BAIAP2//PPP4R2//CBX5//AMFR//PSMC6// |
| GO:0030674 | protein binding, bridging | Molecular function | 8 | 170 | 178 | 17548 | 4.63925974884336 | 0.000347505571809101 | 0.225183610532297 | 3.45903822765934 | 0.0449438202247191 | VAV3//ARHGAP1//CNTNAP1//BAIAP2//PPP4R2//CBX5//AMFR//PSMC6// |
| GO:0019203 | carbohydrate phosphatase activity | Molecular function | 2 | 10 | 178 | 17548 | 19.7168539325843 | 0.00436444681363093 | 1 | 2.36007079457291 | 0.0112359550561798 | PFKFB3//FBP2// |
| GO:0050308 | sugar-phosphatase activity | Molecular function | 2 | 10 | 178 | 17548 | 19.7168539325843 | 0.00436444681363093 | 1 | 2.36007079457291 | 0.0112359550561798 | PFKFB3//FBP2// |
| GO:0005391 | sodium:potassium-exchanging ATPase activity | Molecular function | 2 | 12 | 178 | 17548 | 16.4307116104869 | 0.00631644406014756 | 1 | 2.19952734571685 | 0.0112359550561798 | ATP1B2//FXYD2// |
| GO:0008556 | potassium-transporting ATPase activity | Molecular function | 2 | 12 | 178 | 17548 | 16.4307116104869 | 0.00631644406014756 | 1 | 2.19952734571685 | 0.0112359550561798 | ATP1B2//FXYD2// |
| GO:0019894 | kinesin binding | Molecular function | 3 | 40 | 178 | 17548 | 7.3938202247191 | 0.00769866093739016 | 1 | 2.11358480704505 | 0.0168539325842697 | KIF18B//LRP8//MAPK8IP1// |
| GO:0035591 | signaling adaptor activity | Molecular function | 4 | 82 | 178 | 17548 | 4.80898876404494 | 0.00968936764640448 | 1 | 2.01370456522358 | 0.0224719101123595 | VAV3//ARHGAP1//CNTNAP1//MAPK8IP1// |
| GO:0008092 | cytoskeletal protein binding | Molecular function | 17 | 908 | 178 | 17548 | 1.84574073157452 | 0.0110116053686987 | 1 | 1.95814936114245 | 0.0955056179775281 | KLHL2//NF2//MYO15A//CHP1//KIF18B//MAP1LC3A//BAIAP2//STMN2//TPPP3//RHOA//LRP8//MAPK8IP1//CAMSAP2//STIM1//ARPC3//TMEM201//CTNNA3// |
| GO:0004879 | nuclear receptor activity | Molecular function | 3 | 49 | 178 | 17548 | 6.03577161201559 | 0.0134359269405443 | 1 | 1.87173236635201 | 0.0168539325842697 | ESRRG//VDR//NR6A1// |
| GO:0098531 | transcription factor activity, direct ligand regulated sequence-specific DNA binding | Molecular function | 3 | 49 | 178 | 17548 | 6.03577161201559 | 0.0134359269405443 | 1 | 1.87173236635201 | 0.0168539325842697 | ESRRG//NR6A1//VDR// |
| GO:0098641 | cadherin binding involved in cell-cell adhesion | Molecular function | 2 | 19 | 178 | 17548 | 10.3772915434654 | 0.0156221842489901 | 1 | 1.80625824440464 | 0.0112359550561798 | BAIAP2//TRIM29// |
| GO:0030215 | semaphorin receptor binding | Molecular function | 2 | 20 | 178 | 17548 | 9.85842696629213 | 0.0172435031742246 | 1 | 1.76337449868066 | 0.0112359550561798 | RIT2//SEMA3F// |
| GO:0005070 | SH3/SH2 adaptor activity | Molecular function | 3 | 57 | 178 | 17548 | 5.1886457717327 | 0.0201229600820502 | 1 | 1.69630813431472 | 0.0168539325842697 | VAV3//ARHGAP1//CNTNAP1// |
| GO:0003707 | steroid hormone receptor activity | Molecular function | 3 | 59 | 178 | 17548 | 5.01275947438583 | 0.0220328747265872 | 1 | 1.65692883483094 | 0.0168539325842697 | ESRRG//NR6A1//VDR// |
| GO:0019003 | GDP binding | Molecular function | 3 | 59 | 178 | 17548 | 5.01275947438583 | 0.0220328747265872 | 1 | 1.65692883483094 | 0.0168539325842697 | RHOA//RAB9B//RAB2A// |
| GO:0048306 | calcium-dependent protein binding | Molecular function | 3 | 59 | 178 | 17548 | 5.01275947438583 | 0.0220328747265872 | 1 | 1.65692883483094 | 0.0168539325842697 | STMN2//CHP1//VAMP2// |
| GO:0016667 | oxidoreductase activity, acting on a sulfur group of donors | Molecular function | 3 | 61 | 178 | 17548 | 4.84840670473384 | 0.024038429083663 | 1 | 1.61909391697664 | 0.0168539325842697 | TXNDC8//TMX1//SRXN1// |
| GO:0099516 | ion antiporter activity | Molecular function | 3 | 61 | 178 | 17548 | 4.84840670473384 | 0.024038429083663 | 1 | 1.61909391697664 | 0.0168539325842697 | SLC24A3//SLC9B1//SLC37A4// |
| GO:0015081 | sodium ion transmembrane transporter activity | Molecular function | 5 | 161 | 178 | 17548 | 3.06162328145718 | 0.0242380031334458 | 1 | 1.61550316271731 | 0.0280898876404494 | SCN4A//ATP1B2//FXYD2//SLC24A3//SLC9B1// |
| GO:0015631 | tubulin binding | Molecular function | 8 | 351 | 178 | 17548 | 2.24693492109222 | 0.0270346121382285 | 1 | 1.56807985680541 | 0.0449438202247191 | CHP1//KIF18B//MYO15A//MAP1LC3A//STIM1//CAMSAP2//STMN2//TPPP3// |
| GO:0019706 | protein-cysteine S-palmitoyltransferase activity | Molecular function | 2 | 27 | 178 | 17548 | 7.30253849354973 | 0.0304179660880838 | 1 | 1.51686982862138 | 0.0112359550561798 | ZDHHC19//ZDHHC20// |
| GO:0019707 | protein-cysteine S-acyltransferase activity | Molecular function | 2 | 27 | 178 | 17548 | 7.30253849354973 | 0.0304179660880838 | 1 | 1.51686982862138 | 0.0112359550561798 | ZDHHC19//ZDHHC20// |
| GO:0015491 | cation:cation antiporter activity | Molecular function | 2 | 30 | 178 | 17548 | 6.57228464419476 | 0.0369620659270147 | 1 | 1.43224376267845 | 0.0112359550561798 | SLC24A3//SLC9B1// |
| GO:0016462 | pyrophosphatase activity | Molecular function | 15 | 892 | 178 | 17548 | 1.65780722527334 | 0.0380320439317068 | 1 | 1.41985033395185 | 0.0842696629213483 | KIF18B//DNAH12//MYO15A//ARF3//RHOA//RAB9B//RAB2A//RIT2//GNA14//DDX55//LHPP//ATP1B2//FXYD2//PSMC6//ABCD3// |
| GO:0030276 | clathrin binding | Molecular function | 3 | 73 | 178 | 17548 | 4.05140834231184 | 0.0380687198520411 | 1 | 1.41943172708745 | 0.0168539325842697 | SYT2//TOM1L2//DOC2A// |
| GO:0016817 | hydrolase activity, acting on acid anhydrides | Molecular function | 15 | 894 | 178 | 17548 | 1.6540984842772 | 0.0386635848402603 | 1 | 1.41269788118782 | 0.0842696629213483 | KIF18B//DNAH12//MYO15A//ARF3//RHOA//RAB9B//RAB2A//RIT2//GNA14//DDX55//LHPP//ATP1B2//FXYD2//PSMC6//ABCD3// |
| GO:0016818 | hydrolase activity, acting on acid anhydrides, in phosphorus-containing anhydrides | Molecular function | 15 | 894 | 178 | 17548 | 1.6540984842772 | 0.0386635848402603 | 1 | 1.41269788118782 | 0.0842696629213483 | KIF18B//DNAH12//MYO15A//ARF3//RHOA//RAB9B//RAB2A//RIT2//GNA14//DDX55//LHPP//ATP1B2//FXYD2//PSMC6//ABCD3// |
| GO:0016417 | S-acyltransferase activity | Molecular function | 2 | 31 | 178 | 17548 | 6.36027546212396 | 0.0392529926444491 | 1 | 1.40612722708624 | 0.0112359550561798 | ZDHHC19//ZDHHC20// |
| GO:0098632 | cell-cell adhesion mediator activity | Molecular function | 2 | 31 | 178 | 17548 | 6.36027546212396 | 0.0392529926444491 | 1 | 1.40612722708624 | 0.0112359550561798 | BAIAP2//TRIM29// |
| GO:0015298 | solute:cation antiporter activity | Molecular function | 2 | 34 | 178 | 17548 | 5.7990746860542 | 0.0464361112698019 | 1 | 1.33314415682279 | 0.0112359550561798 | SLC24A3//SLC9B1// |
| GO:0043178 | alcohol binding | Molecular function | 3 | 80 | 178 | 17548 | 3.69691011235955 | 0.0478036040357266 | 1 | 1.32053935958402 | 0.0168539325842697 | STARD3//SYT2//CDIPT// |
| GO:0017111 | nucleoside-triphosphatase activity | Molecular function | 14 | 846 | 178 | 17548 | 1.6314181740909 | 0.0493579056632444 | 1 | 1.30664327640984 | 0.0786516853932584 | KIF18B//DNAH12//MYO15A//ARF3//RHOA//RAB9B//RAB2A//RIT2//GNA14//DDX55//ATP1B2//FXYD2//PSMC6//ABCD3// |
